# Pattern of outpatient utilisation and cost for patients under the Universal Coverage

**DOI:** 10.1186/1471-2458-14-S1-P9

**Published:** 2014-01-29

**Authors:** Kanet Sumputtanon, Nilawan Upakdee, Pudtan Phanthunane, Supasit Pannarunothai

**Affiliations:** 1 Health Systems Research Institute, Nonthaburi 11000, Thailand; 2 Faculty of Pharmaceutical Sciences, Naresuan University, Phitsanulok 65000, Thailand; 3 Faculty of Business, Economics and Communications, Naresuan University, Phitsanulok 65000, Thailand; 4 Faculty of Medicine, Naresuan University, Phitsanulok 65000, Thailand

## Background

Universal Coverage (UC) is a public health insurance scheme covered approximately 48.3 million of Thai population (74.3% of total) in 2011. This study aimed to categorise pattern of outpatient utilisation and examine the relationship between an annual cost of outpatient service and pattern of utilisation.

## Materials and methods

Outpatient data from the National Health Security Office in 2011 were analysed, 4 provinces were picked to represent the pattern of service utilisation. The selection criteria emphasised on 1) availability of various types of hospitals in the province (health promoting, community, general/regional, university hospital; and others), and 2) the maximum utilisation rate of people in the province. Descriptive statistical analysis was employed to calculate annual utilisation. One-way analysis of variance was used to calculate the association between the annual costs per person and the pattern of service utilisation (whether rural or urban or both; and the type of hospital).

## Results

Most common single users were at the health promoting hospitals (26.2%-32.3%), followed by community hospitals (17.6%-22.6%) and general/regional hospitals (2.8%-13.9%). For two-service-type users, the most common was the combination of health promoting hospitals and community hospitals (14.1%-23.5%), followed by health promoting and general/regional hospitals (2.3%-7.4%), and community hospitals and general /regional hospitals (1.5%-3.8%). For three-service-type users, most occurred at health promoting, community and general/regional hospitals (2.0%-5.0%). The other patterns were less than 1.0%. All four provinces had a similar pattern of urban (42.8%-52.0%), urban and rural (24.4%-29.8%), and rural only (19.6%-27.9%). Costs per patient per year were significantly different between those using hospital combination and geographical area. Homogeneous subsets test suggested seven utilization patterns.

## Conclusions

Different pattern of outpatient utilisation should be taken into account for policy formulation of capitation payment and allocation criteria.

